# A Multiplex Fluidic Chip for Rapid Phenotypic Antibiotic Susceptibility Testing

**DOI:** 10.1128/mBio.03109-19

**Published:** 2020-02-25

**Authors:** Pikkei Wistrand-Yuen, Christer Malmberg, Nikos Fatsis-Kavalopoulos, Moritz Lübke, Thomas Tängdén, Johan Kreuger

**Affiliations:** a Department of Medical Cell Biology, Uppsala University, Uppsala, Sweden; b Department of Medical Sciences, Uppsala University, Uppsala, Sweden; c Gradientech AB, Uppsala, Sweden; University of Pittsburgh School of Medicine

**Keywords:** antibiotic susceptibility testing, clinical isolates, fluidic chip, microfluidics, multiplex

## Abstract

Many patients with severe infections receive inappropriate empirical treatment, and rapid detection of bacterial antibiotic susceptibility can improve clinical outcome and reduce mortality. To this end, we have developed a multiplex fluidic chip for rapid phenotypic antibiotic susceptibility testing of bacteria. A total of 21 clinical isolates of Escherichia coli, Klebsiella pneumoniae, and Staphylococcus aureus were acquired from the EUCAST Development Laboratory and tested against amikacin, ceftazidime, and meropenem (Gram-negative bacteria) or gentamicin, ofloxacin, and tetracycline (Gram-positive bacteria). The bacterial samples were mixed with agarose and loaded in an array of growth chambers in the chip where bacterial microcolony growth was monitored over time using automated image analysis. MIC values were automatically obtained by tracking the growth rates of individual microcolonies in different regions of antibiotic gradients. Stable MIC values were obtained within 2 to 4 h, and the results showed categorical agreement with reference MIC values as determined by broth microdilution in 86% of the cases.

## Abstract (precis)

Prompt and effective antimicrobial therapy is crucial for the management of patients with severe bacterial infections but is becoming increasingly difficult to provide due to emerging antibiotic resistance. The traditional methods for antibiotic susceptibility testing (AST) used in most clinical laboratories are reliable but slow with turnaround times of 2 to 3 days, which necessitates the use of empirical therapy with broad-spectrum antibiotics. There is a great need for fast and reliable AST methods that enable starting targeted treatment within a few hours to improve patient outcome and reduce the overuse of broad-spectrum antibiotics. The multiplex fluidic chip for phenotypic AST described in the present study may enable data on antimicrobial resistance within 2 to 4 h, allowing for an early initiation of appropriate antibiotic therapy.

## INTRODUCTION

Antibiotics are among the most successful drugs developed and have helped to drastically reduce mortality and morbidity from common bacterial infections such as pneumonia, urinary tract infections, and bloodstream infections over the last century (1). Unfortunately, many antibiotics are becoming less effective due to the selection and spread of antibiotic-resistant bacteria (2–4), partly resulting from antibiotic overuse (5). The increasing resistance to standard antibiotic therapy increases the risk of inappropriate empirical antibiotic therapy, treatment failure, and mortality in critically ill patients (6). For example, carbapenem resistance is increasing in Europe, and in Greece, carbapenem resistance in Klebsiella pneumoniae is already approaching 50% incidence in the hospital setting (7, 8). Thus, new rapid and robust methods for antibiotic susceptibility testing (AST) that can deliver data on bacterial antibiotic resistance within hours instead of days are much needed. Such methods will make it possible to prescribe targeted antibiotic therapy, which will improve clinical outcome and reduce unnecessary use of broad-spectrum antibiotics, thereby minimizing the risk of adverse events and resistance development (9–11).

The traditional methods for AST currently used in most clinical microbiology laboratories are reliable and inexpensive but commonly comparatively slow with turnaround times (TATs) of 2 to 3 days (12). Recently, progress has been made to decrease TATs, partly through automation of existing methods but also as a result of the implementation of new technologies (12). For example, recent developments of the rapid disc diffusion test with new breakpoints (4-, 6-, and 8-h readouts) developed by EUCAST allow for TATs of around 24 h, although not for all samples (13). Importantly, studies show that in severe infections such as septic shock and bacterial meningitis, delayed appropriate antibiotic treatment is associated with increased mortality (10).

Many new rapid AST methods under development are phenotypic tests, as genotypic tests for selected resistance markers currently cannot reliably predict antibiotic susceptibility due to the complexity and rapid evolution of the bacterial resistome (9, 12, 14). The latest developments in resistance screening methods include mass spectrometry to identify resistance-associated proteins and degradation products of antibiotics, as well as PCR-based techniques, RNA microarrays, and whole-genome sequencing together with artificial intelligence-based deep learning tools to detect resistance markers (14, 15). Novel microfluidic systems for phenotypic antibiotic susceptibility testing are also being developed by several research labs (16).

We have previously designed and evaluated a rapid phenotypic antibiotic susceptibility test where bacteria are mixed with agarose to form a gel within a growth chamber inside a microfluidic system and subjected to a gradient of an antibiotic to enable MIC determination (17). The aim of the current study was to develop a higher throughput system (based on our previous design) capable of simultaneously analyzing eight samples on one chip and with the possibility of testing several antibiotics in parallel. To accomplish this, we developed a microfluidic chip using three-dimensional (3D) printed molds for polydimethylsiloxane (PDMS) casting, together with a 3D printed chip holder with an integrated reservoir lid. Bacterial microcolony growth was monitored using dark-field time-lapse microscopy, and microcolony growth was quantified using a cluster analysis algorithm. MIC values were obtained by analysis of bacterial growth rates in different regions of the antibiotic gradients. In this initial evaluation of the new system, clinical isolates of Escherichia coli, Klebsiella pneumoniae, and Staphylococcus aureus with different antibiotic susceptibility profiles were tested against six commonly used antibiotics, and stable MIC values were obtained within 2 to 4 h.

## RESULTS

### Design and manufacture of the fluidic system.

**FIG 1:**
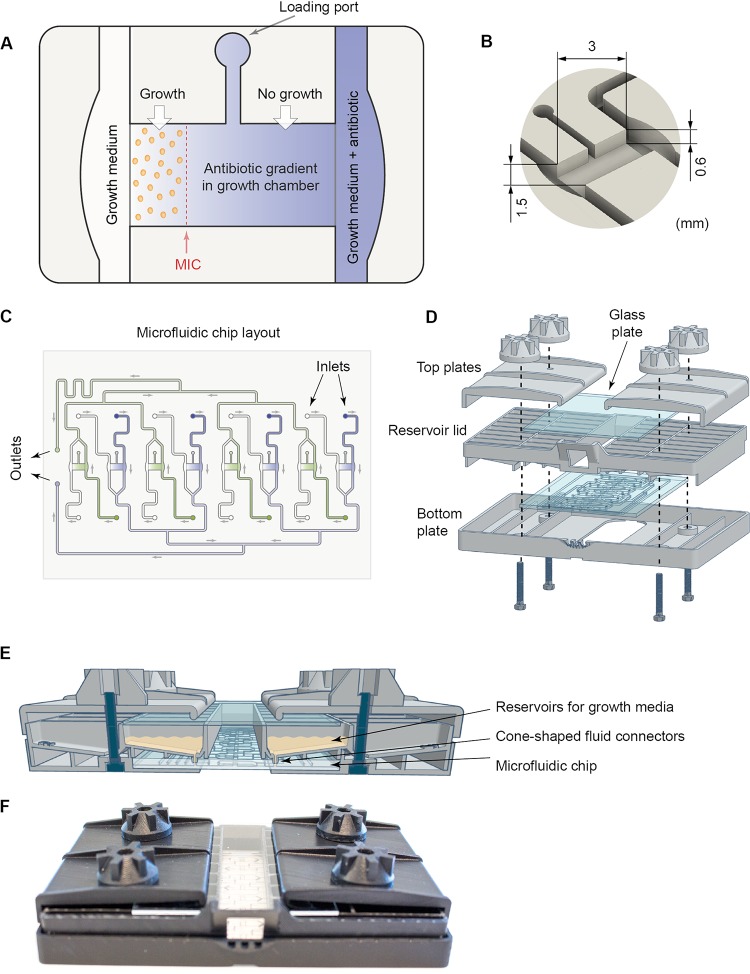
Overview of the fluidic system. (A) Illustration showing a growth chamber and a loading port used to inject the bacterium-agarose mix. The growth chamber is flanked by two flow channels. Antibiotic gradients in the growth chamber are created by diffusion from the source channel (blue) containing growth medium as well as a high concentration of the antibiotic to the sink channel (white) containing only growth medium. Bacteria present in the growth chamber are exposed to the diffusion-limited antibiotic gradient, and the MIC value will correspond to the lowest concentration at which the bacteria do not grow to form microcolonies (shown in this example as a red dashed line). (B) A close-up view of a growth chamber with the dimensions indicated. (C) Overview of the microfluidic chip holding eight growth chambers. The fluidic outlets that are connected to a pump are indicated, as well as the inlets (16 in total) that are connected to the reservoir lid. (D) Drawing showing how the bottom plate, fluidic chip, reservoir lid, and top plates are assembled. (E) Cartoon showing the assembled system from the side highlighting the connections between the reservoir lid and the fluidic chip via fluid connectors. (F) Photograph of the assembled system.

The goal of the present study was to develop a multiplex fluidic system that allows for parallel and automatic analyses of eight independent samples to obtain MIC values for different antibiotics. The construction of the multiplex fluidic system required several new technical solutions. On the fluidic chip, each growth chamber, flanked by two fluidic channels used for delivery of growth media and antibiotics, was designed to feature a loading port that was used to inject bacteria mixed with agarose (Fig. 1A to C). The growth chamber was designed to hold a volume of 5 μl. Loading the bacterium-agarose mix filled up the growth chamber all the way to the edges of the flanking fluidic channels, which were designed to be slightly enlarged at the location of the growth chamber to tolerate slight variations in agarose gel volume (e.g., overloading) without compromising fluid flow. Consistent with our previous study (17), there was no fluid flow through the gel-filled growth chamber. Instead, molecules travel through the growth chamber between the two flanking flow channels by diffusion. Thus, diffusion-limited antibiotic gradients can be established through the agarose gel by the addition of an antibiotic to one of the two flow channels that flank a growth chamber (Fig. 1A). Importantly, the constant fluid flow on both sides of the growth chamber establishes a stable source-sink system. MIC values are then determined by analyzing bacterial microcolony growth in the different regions of the established antibiotic gradient that correspond to distinct antibiotic concentrations (Fig. 1A).

In order to optimally position the eight growth chambers together with their associated flow channels and fluidic inlets and outlets, the growth chambers were divided into two groups that within each group had the same orientation and shared a common outlet channel (Fig. 1C). The chip was generated by casting PDMS into 3D printed molds coding for the fluidic channels and growth chambers. The resultant PDMS chip was then removed from the mold whereafter the PDMS chip was bonded to a glass plate to create a closed fluidic system (see Materials and Methods for a detailed description). The fluidic chip was connected to a 3D printed reservoir lid that was part of a larger chip holder (Fig. 1D to F). The lid had 16 medium reservoirs with small cone-shaped fluid connectors in the bottom, which could be pressed into the chip inlets (Fig. 1E), to enable delivery of growth medium with or without antibiotics from the reservoirs to the different fluidic channels of the system. The reservoir lid was held together with the microfluidic chip, a 3D printed bottom plate and two top plates (including a glass plate positioned above the medium reservoirs to prevent evaporation) by M3 screws passing through the bottom plate, reservoir lid, and top plates to ensure tight fluid connections between the lid reservoirs and the chip (Fig. 1D to F).

### System validation.

**FIG 2:**
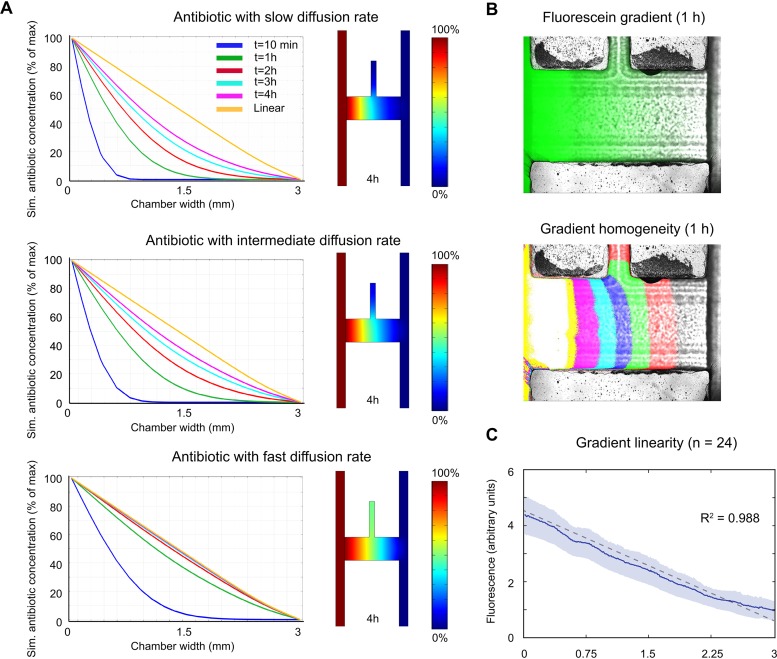
Gradient characterization. (A) Simulations of the formation of antibiotic concentration gradients in the growth chamber, where the antibiotic for the simulation was assigned either a low (slow), intermediate, or high (fast) diffusion rate. The panels to the right show simulations of the concentrations of the respective antibiotics (with different diffusion properties) in the growth chamber and adjacent source and sink flow channels 4 h after onset of gradient generation from maximum (100%) (red) to low (0%) (blue) antibiotic concentration. (B) Gradient formation in the growth chambers was validated using fluorescein. Each colored field in the bottom panel corresponds to a portion of the gradient corresponding to 12.5% of the total gradient. (C) Average fluorescein gradient (solid line) (95% confidence interval indicated by the blue area) and the linear least-squares fit (dashed line) of the data (*n *= 24).

Simulations of gradient formation of antibiotics with different agar diffusion rates in a chip growth chamber were performed using Comsol (see Materials and Methods for details). Three separate simulations were performed where the antibiotics were assigned either a low (slow) diffusion rate based on the rate for the chloramphenicol antibiotic, which has the lowest diffusion coefficient reported in the literature, or an intermediate diffusion rate based on the average of several antibiotic diffusion coefficients, or a high (fast) diffusion rate similar to the rate for the penicillin antibiotic, which has the highest diffusion coefficient reported (see Table S1 and Text S1 in the supplemental material). For the simulations with the intermediate and fast diffusion rates, gradients that were approximately linear were formed after 1 and 3 h, respectively. At these time points, the deviations from linearity (defined as the maximum difference from linearity throughout the chamber) were 13% for the fast diffusion rate and 16% for the intermediate diffusion rate (Fig. 2A). For the slowest diffusion rate, the deviation from linearity at 4 h was 21%.

10.1128/mBio.03109-19.1 TABLE S1 Corrected diffusion coefficients for antibiotics in 0.5% agar used for simulations of gradient formation. Download Table S1, DOCX file, 0.02 MB. Copyright © 2020 Wistrand-Yuen et al. 2020 Wistrand-Yuen et al. This content is distributed under the terms of the Creative Commons Attribution 4.0 International license.

10.1128/mBio.03109-19.2 TEXT S1 Rationale for calculations of corrected antibiotic diffusion coefficients. Download Text S1, DOCX file, 0.02 MB. Copyright © 2020 Wistrand-Yuen et al. 2020 Wistrand-Yuen et al. This content is distributed under the terms of the Creative Commons Attribution 4.0 International license.

Next, a fluorescein dye was used as a model molecule for antibiotic diffusion to visualize and validate the formation of antibiotic gradients in the growth chambers experimentally. The molecular weight and diffusion characteristics of fluorescein are similar to those of small molecule antibiotics (18). The system was operated using a syringe pump (see Materials and Methods for details) and quantifications of the gradients performed after 2 h, when the fluorescein gradients were considered to be fully established. Notably, the homogeneity of the gradients formed in the growth chambers was clearly affected by the loading port channel, which acted as a sink (Fig. 2B). Therefore, the half of the growth chamber closest to the loading port was later excluded from the image analysis of bacterial growth. The gradients formed in the other half of the growth chamber (i.e., distal to the loading port) were shown to be linear (*R*^2^ = 0.988) and consistent between different chambers and chips (Fig. 2C).

### Data acquisition and calculation of MIC values.

**FIG 3:**
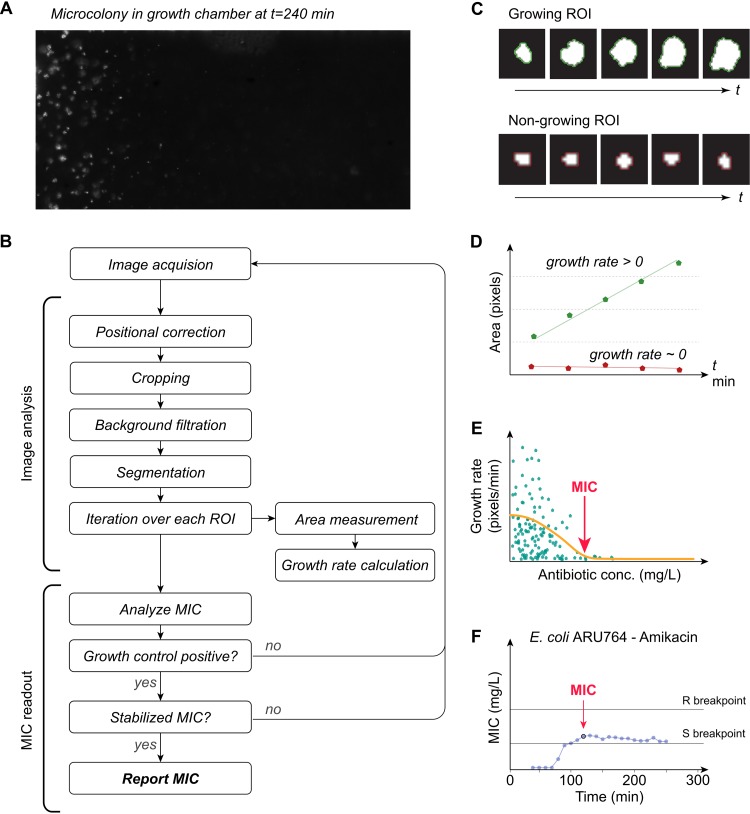
Data analysis. (A) Example showing E. coli grown in a gradient of amikacin. (B) Flow chart summarizing key actions of the image analysis algorithm. (C and D) Bacterial microcolonies that are exposed to antibiotic concentrations below the MIC (growing region of interest [ROI]) keep expanding over time (green line in panel D), whereas colonies exposed to antibiotic concentrations at or above the MIC (nongrowing ROI) show no growth (red line in panel D). (E) Colony growth expressed as a function of antibiotic concentration. MIC values were calculated for every time point. (F) A final MIC value was reported if it had remained stable (± 5%) for 30 min.

An automated dark-field microscope was used to perform time-lapse imaging of every growth chamber in the fluidic chip, and images from the different growth chambers were acquired every 10 min. Figure 3 shows an example where E. coli bacteria were subjected to a gradient of amikacin to illustrate how the data were acquired and handled in order to obtain MIC values. An image analysis algorithm was developed to extract data from the series of images recorded from the growth chambers for identification of bacterial microcolonies, tracking their growth over time (Fig. 3A and B) to automatically determine MIC values (Fig. 3C to F). Briefly, images of the growth chambers were first corrected for mechanical drift. The images were thereafter cropped to include only the portions of the growth chambers where the antibiotic gradients were linear, followed by subtraction of background signal. To be able to quantify growth, each image was binarized so that every colony was represented by a cluster of positive pixels, and the size of the cluster was monitored and compared over time. Bacterial microcolonies unaffected by the local antibiotic concentration initially showed a linear growth pattern, even though the bacteria can be assumed to grow exponentially. This is consistent with previous studies showing that the colony area increase initially is linear or approximately linear for small colonies of bacteria growing on agar plates (19). Nongrowing colonies exhibited a constant size throughout the experiment (Fig. 3C and D), as expected. Colony growth was next expressed as a function of antibiotic concentration, and a Gaussian fit was used to calculate a MIC value at every time point. A final MIC value was reported once the corresponding positive growth chamber had flagged positive and if the calculated MIC value remained stable (±5%) for 30 min (Fig. 3E and F) or alternatively was reported as the value obtained at the very end of the run (at *t *= 300 min).

### Rapid antibiotic susceptibility testing.

After testing of the fluidic system and optimization of the accompanying analysis algorithm, we performed rapid AST of 21 clinical isolates: 11 Gram-negative (G−) strains (6 E. coli strains and 5 K. pneumoniae strains) and 10 Gram-positive (G+) strains (S. aureus). Every strain was tested four times against three antibiotics: amikacin, ceftazidime, and meropenem for the G− strains and gentamicin, ofloxacin, and tetracycline for the G+ strains. A positive-control growth chamber containing bacteria but no antibiotics was included for each replicate (see Fig. S1 in the supplemental material). All MIC values measured in the fluidic chip were compared to reference MICs generated by the broth microdilution (BMD) method (Tables 1 and 2).

**TABLE 1:** Comparison of MIC values obtained for Gram-negative strains using BMD or the fluidic chip assay developed here

Species and strain	MIC (mg/liter) of antibiotic by BMD or FC method^*a*^					
	Amikacin		Ceftazidime		Meropenem	
	BMD	FC	BMD	FC	BMD	FC
E. coli						
ARU764	16	16	≥16	≥16	≥32	4
ARU754	≥64	≥64	≥16	≥16	16	≤1
ARU755	4	≤2	≤0.5	≤0.5	≤1	≤1
ARU756	≤2	≤2	8	≥16	≤1	≤1
ARU757	4	≤2	1	≤0.5	≤1	≤1
ARU758	8	≤2	≤0.5	2	≤1	≤1

K. pneumoniae						
ARU759	4	≤2	≥16	8	4	≤1
ARU760	4	≤2	≥16	≥16	2	≤1
ARU761	≤2	≤2	≤0.5	≤0.5	≤1	≤1
ARU762	≤2	≤2	≤0.5	≤0.5	≤1	≤1
ARU763	≤2	≤2	1	8	≤1	≤1

EA^*b*^		91%		82%		73%

a MIC values were compared using broth microdilution (BMD) as the reference method and the fluidic chip (FC) assay developed here.

b EA, essential agreement.

**TABLE 2:** Comparison of MIC values obtained for Gram-positive strains (S. aureus) using BMD or the fluidic chip assay developed here

S. aureus strain	MIC (mg/liter) of antibiotic by BMD or FC method^*a*^					
	Gentamicin		Ofloxacin		Tetracycline	
	BMD	FC	BMD	FC	BMD	FC
ARU795	0.25	≤0.125	1	1	0.5	≤0.25
ARU796	0.25	≤0.125	2	2	≥8	4
ARU797	0.5	≤0.125	0.5	0.5	0.5	≤0.25
ARU798	0.5	≤0.125	0.5	1	1	≤0.25
ARU799	0.5	≤0.125	2	2	≥8	≥8
ARU800	0.5	0.25	≥4	≥4	2	0.5
ARU801	1	≤0.125	0.5	0.5	0.5	≤0.25
ARU802	1	0.25	1	1	≤0.25	≤0.25
ARU803	2	1	0.5	≤0.125	≤0.25	≤0.25
ARU804	2	≤0.125	≥4	≥4	4	≤0.25

EA^*b*^		40%		90%		70%

a MIC values were compared using broth microdilution (BMD) as the reference method and the fluidic chip (FC) assay developed here.

b EA, essential agreement.

10.1128/mBio.03109-19.4 FIG S1 Summary of raw data (reported MIC values over time) for all strains analyzed. Download FIG S1, DOCX file, 0.9 MB. Copyright © 2020 Wistrand-Yuen et al. 2020 Wistrand-Yuen et al. This content is distributed under the terms of the Creative Commons Attribution 4.0 International license.

**FIG 4:**
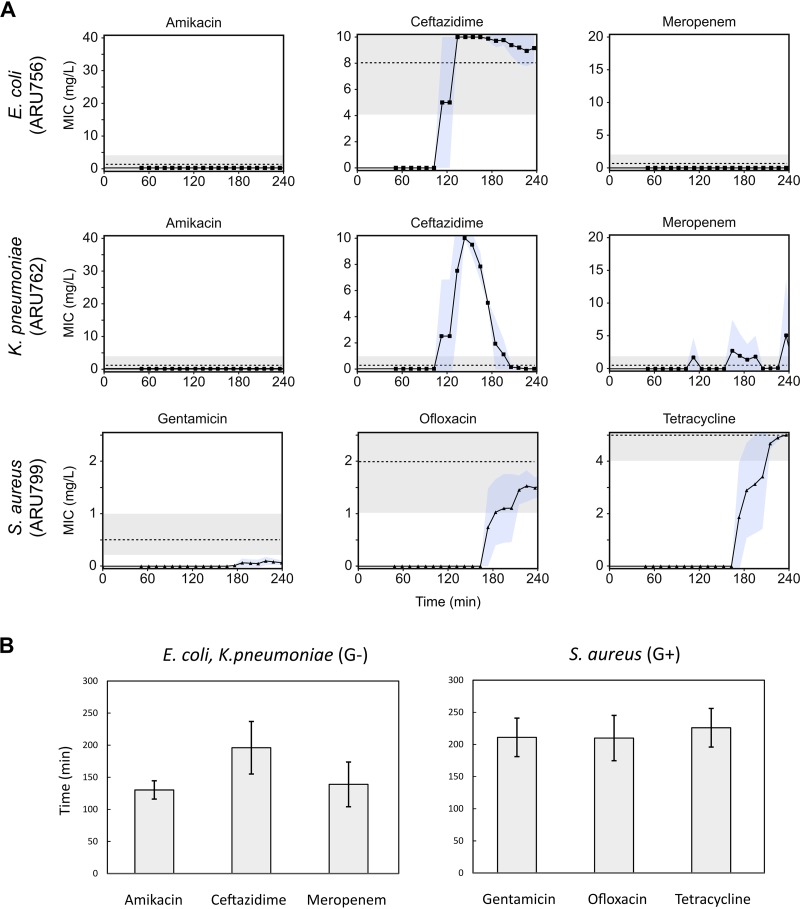
Examples of MIC values. (A) Data from representative strains of E. coli, K. pneumoniae, and S. aureus detailing the MIC signal response over time for amikacin, ceftazidime, and meropenem (E. coli and K. pneumoniae) and gentamicin, ofloxacin, and tetracycline (S. aureus). Blue shading depicts standard deviations (SD) (*n *= 4). The dotted lines correspond to the reference BMD MIC values, where visible in the tested concentration range. Gray shading shows acceptable variation of BMD, ±1 log_2_ dilution. (B) Average readout times until a stable MIC value after start of analysis for Gram-negative (G−) and Gram-positive (G+) bacteria. Error bars depict SD.

Figure 4 shows the mean MIC calculations over time for four replicates and three representative strains (data including repeatability [standard deviations {SD}] for all strains is shown in Table S2). In the examples shown of representative strains of E. coli (ARU756) and K. pneumoniae (ARU762), both amikacin and meropenem MICs were below the limit of quantification (LOQ) throughout the experiment (Fig. 4). However, when the strains were exposed to ceftazidime, they initially appeared to grow unhindered for the first 120 min, followed by a drop in the MIC value. In E. coli ARU756, a ceftazidime-resistant strain, the MIC decreased only slightly, and the resulting MIC was within the range compared to the BMD MIC. For K. pneumoniae ARU762, a ceftazidime-susceptible strain, a more apparent drop in MIC from resistant to susceptible below the LOQ was observed. Similar false-positive peaks were evident in several strains sensitive to ceftazidime and were likely due to phenotypic responses resulting in morphological changes such as filamentation. Filamentation is a well-known phenotypic effect of PBP3-targeting beta-lactams like ceftazidime (20, 21).

10.1128/mBio.03109-19.3 TABLE S2 Summary of time to MIC and MIC values for all strains analyzed. Download Table S2, DOCX file, 0.05 MB. Copyright © 2020 Wistrand-Yuen et al. 2020 Wistrand-Yuen et al. This content is distributed under the terms of the Creative Commons Attribution 4.0 International license.

For the antibiotics tested against G− isolates, 100% categorical agreement (CA) and 91% essential agreement (EA) were observed for amikacin, 82% CA and 82% EA for ceftazidime, and 74% CA and 74% EA for meropenem (Tables 1, 2, and 3). For the antibiotics tested against G+ isolates, the agreement was 80% CA and 40% EA, 100% CA and 90% EA, and 80% CA and 70% EA for gentamicin, ofloxacin, and tetracycline, respectively. The overall categorical agreement between the new fluidic chip and BMD was 85.6%, with a range from 67 to 100%, and very major error (VME) (when resistant strains are misclassified as susceptible), major error (ME) (when susceptible strains are misclassified as resistant), and minor error (MiE) (where resistant or susceptible strains are misclassified as sensitive with increased exposure or strains that were sensitive with increased exposure were misclassified as susceptible or resistant) were 26.2, 3.7, and 9%, respectively (Table 3).

**TABLE 3:** Categorical agreement between BMD tests and the fluidic chip assay described here^*a*^

Species	Antibiotic^*b*^	No. of strains in the following category^*c*^:			No. (%) of agreement results			
		S	I	R	CA	ME	VME	MiE
*E. coli* (*n* = 6)	AMI	4 (4)	1(1)	1 (1)	6 (100)	0 (0)	0 (0)	0 (0)
	CAZ	2 (3)	1 (0)	3 (3)	5 (83)	0 (0)	0 (0)	1 (17)
	MER	5 (4)	1 (0)	0 (2)	4 (67)	0 (0)	1 (17)	1 (17)
*K. pneumoniae* (*n =* 5)	AMI	5 (5)	0 (0)	0 (0)	5 (100)	0 (0)	0 (0)	0 (0)
	CAZ	2 (3)	0 (0)	3 (2)	4 (80)	1 (20)	0 (0)	0 (0)
	MER	5 (4)	0 (1)	0 (0)	4 (80)	0 (0)	0 (0)	1 (20)
*S. aureus* (*n* = 10)	GEN	10 (8)	0 (0)	0 (2)	8 (80)	0 (0)	2 (20)	0 (0)
	OFL	6 (6)	0 (0)	4 (4)	10 (100)	0 (0)	0 (0)	0 (0)
	TET	8 (6)	0 (1)	2 (3)	8 (80)	0 (0)	1 (10)	1 (10)
								
Total		47 (43)	3 (3)	13 (17)	54 (85.6)	1 (1.6)	4 (6.3)	4 (6.3)

a Categorical agreement (CA) was scored according to the EUCAST S-I-R classification (where S is susceptible, I is susceptible with increased exposure, and R is resistant). Total categorical agreement between the MIC values obtained by the new fluidic chip and reference BMD method was 85.6%, with a range from 67 to 100%, and very major (VME), major (ME), and minor error (MiE) rates were 6.3, 1.6, and 6.3%, respectively.

b AMI, amikacin; CAZ, ceftazidime; MER, meropenem; GEN, gentamicin; OFL, ofloxacin; TET, tetracycline.

c Number of strains categorized in the S, I, and R categories according to the fluidic chip assay. The number of strains in these categories by BMD (according to EUCAST clinical breakpoint table version 9.0) are shown within parentheses.

The time to results were on average 155 min (SD, 43 min) for G− isolates (*n *= 132) and 216 min (SD, 32 min) for G+ isolates (*n *= 120), consistent with a higher growth rate of G− strains (Fig. 4B).

## DISCUSSION

The fluidic chip method presented here is able to rapidly provide antibiotic susceptibility data for up to eight samples simultaneously, within 2 to 4 h after the start of measurement in 89% of the tests and within 5 h for 100% of the tests. This is significantly shorter than traditional diagnostics such as disc diffusion and BMD which requires 18 to 24 h. The fluidic chip method provides high information density per test chamber due to the analysis of bacteria growing in antibiotic gradients and has higher resolution than reference BMD tests and similar methods, which are usually based on discrete concentration steps dispersed over a logarithmic (log_2_) scale. When comparing the results obtained using the fluidic chip method described here to BMD, it is important to keep in mind that the present pilot study used a relatively small selection of isolates (*n *= 21) with the aim of providing initial proof-of-concept data for the method.

The comparatively narrow antibiotic concentration range is the main disadvantage of this new method, and inevitably, many strains will have a MIC outside the linear interval. These strains are then reported as MIC values below LOQ or above the maximum concentration, similar to the disc diffusion test, which also has a narrower concentration range than the BMD does. Notably for the method presented, the linear gradient was positioned to include the susceptible (S), susceptible with increased exposure (I), and resistant (R) breakpoints (EUCAST) for each antibiotic tested, as well as one log_2_ dilution step above the R breakpoint and below the S breakpoint, which is the range that arguably is of highest clinical importance. However, thee narrow antibiotic concentration ranges investigated compared to reference BMD data are in line with previous comparative studies of AST methods (22, 23). On the other hand, the main advantage of using a linear gradient is that the resolution is higher in the clinically important range, with significantly lower variation than BMD, as seen in Fig. 4.

The problem of covering a relatively narrow antibiotic concentration range in a single growth chamber can be overcome by testing multiple and complementary antibiotic gradients in parallel growth chambers. Other ways to extend the investigated antibiotic concentration range could be to design growth chambers with nonsymmetrical shape in order to produce nonlinear gradients (24) (similar to the logarithmic [log_2_] dilution steps used in BMD), although this will alter and decrease resolution. Another alternative to increase the antibiotic concentration range tested in a single growth chamber could be to increase the growth chamber width; this would however also increase the time it takes for the gradient to be established and thereby increase the time to results. Taken together, we believe that the design of the system presented is a good compromise in order to achieve acceptable resolution over a relatively wide antibiotic concentration range and at a speed that is much faster than the currently available conventional assays.

For some drugs, there are significant differences in the antibiotic concentration range needed for efficient AST testing depending on the bacterial species, for example for the EUCAST breakpoints for gentamicin against S. aureus (R > 1) and enterococci (R > 128). To address this challenge, it is of course possible to have two or more growth chambers with gradients spanning different concentration ranges of the same antibiotic. Indeed, since the method is intended to be used before the species identity is known, the panel has to be broad enough to support multiple species.

The performance of the current system is dependent on the formation of stable, repeatable antibiotic concentration gradients in multiple parallel growth chambers. Stable gradients are formed over time in a very reproducible manner by diffusion from the antibiotic source flow channel through the agarose gel to the sink channel. The gradient equilibration time depends on the diffusion constant of the antibiotic, which is influenced by temperature, the agarose gel concentration, and the charge, shape, and molecular weight of the antibiotic. For diffusion through agarose, the molecular weight of the antibiotic is the most important parameter to affect diffusion (25). On the basis of our theoretical simulations of gradient formation (Fig. 2), the gradient in the growth chamber will be approximately linear within 1 to 3 h for the antibiotics tested here.

Another factor affecting the performance of the system is of course the growth speed of the bacteria. Admittedly, the risk for incorrect results could be higher in cases where antibiotics that diffuse slowly in the agarose gel are tested against fast-growing bacteria, which could lead to false-negative results. However, and from a practical point of view, in the system presented, the readout MIC curve is not stable as long as the gradient linearity is not established. Therefore, by waiting until the MIC value has become stable over three consecutive measurement points, the time it may take for some antibiotics to generate a stable gradient in the agarose gel does not impact data quality, but it does indeed take longer to achieve a result. For very slowly diffusing antibiotics, such as vancomycin (17, 26), the agarose concentration can also be lowered (to, for example, 0.25% [wt/vol]) in order to allow faster formation of stable gradients as previously shown (17).

The performance of phenotypic rapid AST methods is clearly dependent on the biological characteristics of the isolate. Slower-growing bacteria will need relatively longer measurement times, no matter the technology used to measure growth. For example, one of the S. aureus isolates included in this study (ARU803) grew very slowly and was the only strain where a MIC value could not be detected within 4 h for any of the tested antibiotics. Second, the type of antibiotic affects the results, since the specific mode of action of an antibiotic will result in different lag times before measurable phenotypic effects can be detected. For example, ceftazidime had a long lag phase of ∼2 h before apparent growth stopped in susceptible bacteria (Fig. 4). Consequently, the analysis algorithm tended to call the ceftazidime MIC values too early and therefore to classify most isolates as resistant, even those that were sensitive. However, by using information on growth patterns provided by the antibiotic-free growth control chamber, and by delaying the time until readout for ceftazidime, this problem could be avoided. Furthermore, the type of resistance mechanism at play can also affect the results, as is likely the case for meropenem in this study where no growth or very weak growth of the two resistant isolates included was detected within 4 h. However, when these samples were run for a longer period of time, bacterial growth was detected (data not shown).

All rapid phenotypic AST methods will face the above-discussed biological challenges to some extent, and whether a specific method will turn out to be viable will hinge on whether the benefit of the rapid readouts outweighs the increase in false or unclear readouts as well as the increased cost (9). Thus, rapid phenotypic AST methods will likely never completely replace traditional AST testing, but instead serve as an important addition to be used for critically ill patients for example (12).

In recent years, several microfluidics-based phenotypic rapid AST methods have been described in the literature, based on either direct or indirect growth markers (27). One such system that has reached the clinical diagnostic market is the Accelerate Pheno system. However, previous methods often suffer from complicated sample preparation and loading procedures and often require pure cultures and sometimes have limitations as to which bacterial strains can be analyzed (27). Many methods may also require complicated and expensive analysis instruments (27), or the use of indirect markers (28), and may lack population scale data (29), or have a limited resolution of the answer (30), or are actually not very rapid in comparison to traditional diagnostics (31, 32). Further, several emerging methods measure the effect on bacterial growth at a single antibiotic concentration (commonly set at the S or R breakpoint), thereby limiting the resolution and clinical utility; or need multiple parallel wells for discrete antibiotic concentrations, which increases the complexity and cost. The Pheno system in particular is limited to a single concentration per antibiotic and uses morphokinetic analysis to estimate a quantitative MIC value based on a database of antibiotic responses for various pretested clinical isolates and concentrations, which means that the quality of the result is dependent on the quality of the database. In contrast, the fluidic system presented in this study is easy to use and robust and provides direct growth data on bacteria growing in an antibiotic gradient that effectively corresponds to a large number of antibiotic concentrations. One disadvantage of the current system is the throughput per chip, as a single chip holds eight growth chambers. This limitation in throughput can however be circumvented by running several chips in parallel, provided that there is capacity to image several chips at the same time. In summary, we conclude that the method presented in this study has the potential to provide very rapid antibiotic susceptibility results of up to eight samples and antibiotics per chip, which potentially allows an earlier switch to appropriate targeted therapy. A shorter time to effective treatment can improve survival in critically ill patients and promote the use of narrow-spectrum antibiotics, thereby lowering the risk of side effects and resistance development.

## MATERIALS AND METHODS

### Construction of the microfluidic system.

The molds for polydimethylsiloxane (PDMS) chip casting, as well as the reservoir lid and the bottom plate, were generated using a Form 2 three-dimensional (3D) printer (Formlabs, Somerville, MA, USA) and printed horizontally using Black Resin (Formlabs) and 50-μm-thick layers (stereolithography [STL] files for 3D printing available on request). The microfluidic chips were generated by casting PDMS (Sylgard 184; Sigma-Aldrich) into 3D printed molds without coating agent. The resultant PDMS chips were bonded to glass plates (62 by 82 by 1 mm) (Marienfeld Superior; Paul Marienfeld GmbH & Co, Germany) with a calibrated corona treater (BD20-AC; ETP, Chicago, IL, USA) essentially as previously described (33).

### Validation of gradient formation.

Simulations of gradient formation were performed using Comsol Multiphysics 5.4 (Comsol AB, Stockholm, Sweden). A nonconstrained Multiphysics 2D model with a shallow channel 3D geometry approximation was used, and the flow used in the model represented the steady-state flow conditions in the system. A nonconstrained interface between the water medium of the flow channels and the agar present in a growth chamber was included to allow for free diffusion of chemical compounds in and out of the chamber. Any losses of water vapor as well as nonhomogeneous geometry of the agar at the inlet port were neglected. Diffusion constants were taken from literature and adjusted to compensate for the density of the agarose gel used in the current study (see Table S1 and Text S1 in the supplemental material).

For the experimental part, fluid flow was created in the fluidic chip using a syringe pump (Chemyx Fusion 200; Chemyx, Stafford, TX, USA) set in withdrawal mode, and a flow rate of 1 μl/min was generated in the chip flow channels for all experiments. The fluorescent dye fluorescein (catalog no. 46955; Sigma-Aldrich) was used to visualize gradient formation, and images of the formed gradients were captured using an Axiovert 200M fluorescence microscope (Carl Zeiss, Jena, Germany). The quantification of the gradient was done using ImageJ1.8.0 (NIH, Bethesda, MD, USA), and the values were normalized to the highest signal in the source channel. Linear regression was conducted using linear square fitting, and standard deviations (SD) were calculated using GraphPad Prism version 6 (GraphPad Software, San Diego, CA, USA).

### Bacterial isolates.

All bacterial strains were provided by the EUCAST Development Laboratory in Växjö, Sweden. Isolates of E. coli (*n* = 6), K. pneumoniae (*n* = 5), and S. aureus (*n* = 10) with different degrees of susceptibility against the tested antibiotics were used in this study (Tables 1 and 2). All strains were cultivated using cation-adjusted Müller-Hinton (MH-II) broth and MH-II agar. Strains were restreaked on agar plates from frozen stocks on a weekly basis.

### Antibiotics.

Antibiotics were purchased from Sigma-Aldrich; ceftazidime and ofloxacin were the European Pharmacopeia standards, and the meropenem used was the U.S. Pharmacopeia standard. All antibiotics were first prepared as 20× stock solutions and stored at –80°C until use. The antibiotic concentrations used in the experiments in the source solutions for gradient generation were 2.5-fold higher than the clinical breakpoints for resistance for the tested antibiotic against the tested specimen in each specific case according to the EUCAST clinical breakpoints table version 9.0 (34). Amikacin, ceftazidime, and meropenem were tested against E. coli and K. pneumoniae using a final source concentration of 40, 10, and 20 mg/liter, respectively, while gentamicin, ofloxacin, and tetracycline were tested against S. aureus using a final concentration of 2.5, 2.5, and 5 mg/liter, respectively. Prior to the start of an experiment, the antibiotics were thawed and diluted in growth medium (MH-II broth; Becton Dickinson).

### Antibiotic susceptibility testing.

Both the growth medium (MH-II broth) and the fluidic chip were degassed for 1 h prior to every experiment to minimize the risk of the formation of gas bubbles within the fluidic system. Suspensions of bacteria were prepared by dissolving two to four colonies in physiological NaCl (0.9% [wt/vol]), and then the suspension was adjusted to 0.5 McFarland. Next, the suspension was further diluted 1:50 in growth medium and mixed 1:1 with 1% agarose (TopVision low-melting-point agarose; Fisher Scientific). Each chamber in the fluidic chip was loaded with 5 μl of the bacterium-agarose mixture and incubated at 4°C for 5 min to solidify the agarose gel. The chip was connected to the reservoir lid and assembled with the bottom plate and the top plates using M3 screws. Finally, tubing was inserted into the two fluid outlets on the chip and connected to two separate syringes fitted in a syringe pump set to produce a flow of 1 μl/min in the fluidic channels on each side of the growth chamber. The chip was primed with growth medium prior to every experiment, and excess liquid removed from the lid reservoirs before growth medium with or without antibiotics was added to the appropriate reservoirs. The fully assembled chip was placed in a custom-built dark-field microscope fitted with a motorized camera module and a circuit board with heat elements. All experiments were conducted at 37°C. Images of each growth chamber were taken every 10 min using a Basler ACE camera (Basler ace acA2500-14uc color; Basler). Data on broth microdilution (BMD) testing was obtained from the EUCAST Development Laboratory (EDL) in Växjö, Sweden.

### Image and data analyses.

Image data were analyzed in Python 2.7.10 using python libraries scipy (version 0.16.1), numpy (version 1.9.3), pandas (version 0.17.0), matplotlib (version 1.5.0rc3), and skimage (version 0.11.3). Image stacks were sorted based on chamber and read as 8-bit images (1,942 by 2,590 pixels). Positioning deviations were corrected using convolution, and the images were cropped to contain only the actual chamber. To reduce background noise, a Gaussian filter was applied to the first image in the stack, and the resultant background image was subtracted from the following images in the stack. Binary images were made using a threshold value of 10 units. Starting from the fifth image, the area of each microcolony was measured and compared to the previous four images in the stack. The growth rate of each microcolony was computed by fitting data to a linear function, where the slope indicates the rate of growth. Growth rates of ≤0 or >100 were likely due to noise and therefore excluded. The chamber was divided into 500 equally spaced bins (approximately 5 pixels/bin), and the microcolony regions of interest were divided into different bins based on their *x* coordinate. The mean of each bin was computed, and data were fitted into a Gaussian distribution. The MIC was read at the first position where the Gaussian distribution < 1. After the MIC signal over time had stabilized and three values in a row were within 5% variation, the MIC was considered stable and was reported. Accordingly, the limit of quantification (LOQ) was defined as 1/20 of the maximum antibiotic concentration. For chambers with no signal, a read-out was performed 30 min after growth was detected in the positive growth control chamber. No MIC values were accepted before growth had been detected in the positive growth control chamber.

For comparison of essential agreement with reference BMD MICs, the obtained median MIC values for four replicates were rounded up to the nearest log_2_ value, to allow for comparison on the same scale. As per the method used for previous comparisons of methods with different concentration ranges (22), when the reference method showed results below or above LOQ for the comparative method, these were counted as in agreement. For comparison of categorical agreement, the MIC values from the essential agreement analysis were categorized by applying the EUCAST breakpoints for susceptible (S), sensitive with increased exposure (I), and resistant (R) categories (The European Committee on Antimicrobial Susceptibility Testing, breakpoint tables for interpretation of MICs and zone diameters, version 9.0, 2019, see http://www.eucast.org/clinical_breakpoints/). Compared to BMD, S instead of R was counted as a very major error (), R instead of S as a major error (ME) and S or R instead of I (or I instead of S or R) as a minor error (MiE).
