# Supplementary material for: A Multiplex Fluidic Chip for Rapid Phenotypic Antibiotic Susceptibility Testing
Source: mBio. 2020 Feb 25;11(1):e03109-19. doi: 10.1128/mBio.03109-19 (PMC7042698; doi:10.1128/mBio.03109-19)
Supplement: TABLE S1 [file mBio.03109-19-st001.docx]

# Table S1. Corrected diffusion coefficients for antibiotics in 0.5% agar used for simulations of gradient formation.

|  |  | D_corr_ (mm2/h) | D (mm2/h) | Agar concentration (%) | MW | Reference |
| --- | --- | --- | --- | --- | --- | --- |
| Levofloxacin/ofloxacin | | 4.13 | 2.064 | 1.5 | 361 | (1) |
| Vancomycin | | 1.80 | 0.72 | 1.7 | 1449 | (2) |
| Teicoplanin | | 1.18 | 0.47 | 1.7 | 1564 | (2) |
| Cefsulodin |  | 1.58 | 0.4104 | 2 | 533 | (3) |
| Chloramphenicol base | | 1.32 | 0.342 | 2 | 323 | (3) |
| Chloramphenicol succinate | | 0.93* | 0.2412 | 2 | 423 | (3) |
| Metronidazole | | 1.66 | 0.432 | 2 | 171 | (3) |
| Piperacillin | | 1.22 | 0.3168 | 2 | 517 | (3) |
| Ciprofloxacin | | 2.05 | 0.54 | 2 | 331 | (4) |
| Penicillin |  | 6.10* | 1.6 | 2 | 349 | (5) |
| Average |  | 2.2* |  |  |  |  |
| * used for simulations  **References:** | | | | | | |

1. Dai H, Wu J, Wang Y, Tan S, Liang S, Jiang B, Zhao N, Xu J. 2011. Diffusion of levofloxacin mesylate in agarose hydrogels monitored by a refractive-index method. Journal of Applied Polymer Science 122:3000–3006.

2. Cavenaghi LA, Biganzoli E, Danese A, Parenti F. 1992. Diffusion of teicoplanin and vancomycin in agar. Diagn Microbiol Infect Dis 15:253–258.

3. Meulemans A, Paycha F, Hannoun P, Vulpillat M. 1989. Measurement and clinical and pharmacokinetic implications of diffusion coefficients of antibiotics in tissues. Antimicrob Agents Chemother 33:1286–1290.

4. Shaw L, Phung C, Grace M. 2015. Pharmaceuticals and personal care products alter growth and function in lentic biofilms. Environ Chem 12:301–306.

5. Humphrey JH, Lightbown JW. 1952. A General Theory for Plate Assay of Antibiotics with some Practical Applications. Microbiology, 7:129–143.
