# Supplementary material for: A Multiplex Fluidic Chip for Rapid Phenotypic Antibiotic Susceptibility Testing
Source: mBio. 2020 Feb 25;11(1):e03109-19. doi: 10.1128/mBio.03109-19 (PMC7042698; doi:10.1128/mBio.03109-19)
Supplement: TEXT S1 [file mBio.03109-19-s0001.docx]

**Text S1.** Rationale for calculations of corrected antibiotic diffusion coefficients.

The simulations presented in Figure 2 were carried out using the diffusion coefficient (D) values (marked by *) in Table S1 corrected for the agar gel concentration used in the growth chambers in the current study. These were calculated according to the following method:

The diffusion of a molecular species through a medium can be described by Fick’s law, which states that the rate of diffusion dn/dt (diffusive flux) across an area A is:

dn/dt = F = –DA∂c/∂x

in which D is the diffusion coefficient, ∂c/∂x is the concentration gradient of the molecule, and dn/dt is the amount of molecules crossing the area A per unit time. A higher D leads to a higher diffusive flux.

In an ideal case, D is constant for a specific molecular species in a specific medium and at a set temperature. Experimentally measured diffusion coefficients for antibiotics in agar gels are difficult to find in literature, but some do exist. The coefficients are usually measured using a 1.5-2% agar concentration (common for agar plates).

In the present study, 0.5% agarose was used in the growth chambers, which leads to more rapid antibiotic diffusion rates as compared to most previous studies. Similarly to the approach in the previous investigation by Hou et al (1), the diffusion coefficients in 0.5% agarose can be estimated by using the Stokes-Einstein approximation,

D = kT/6πrη (Eq. S1)

in which k is the Boltzmann constant, T is the temperature, r is the hydrodynamic radius of the molecule, and η is the viscosity of the medium. By rearranging, we get

Dη/T = k/6πr (Eq. S2)

We observe that k/6πr only contains constants, and therefore is a constant value for a specific molecule. If we assign the constant C:

C = k/6πr (Eq. S3)

Then

D = CT/η (Eq. S4)

Therefore, increasing the viscosity η decreases D, while increasing temperature increases D. If T is kept constant, D depends directly and linearly only on viscosity η. Notably, previous measurements of diffusion coefficients in agar were often conducted at room temperature, while the present fluidic system was run at 37ºC. Since increasing temperature increases diffusion rate, we do not have to compensate for an increased T in these calculations, as an increased T only will lead to faster gradient formation, which is beneficial for the purpose of establishing an antibiotic gradient in the growth chamber as fast as possible.

However, based on experimental studies, changing from a 2% to a 0.5% w/v agarose gel results in a reduction of the viscosity of ~26%, changing from a 1.7% to a 0.5% w/v agarose gel results in a reduction of the viscosity of ~40%, and changing from a 1.5% to a 0.5% w/v agarose gel results in a reduction of the viscosity of ~50% (2). Hence, we can calculate a corrected diffusion coefficient D_corr_ in 0.5% gel:

D_corr_ = D / η_fraction_ (Eq. S5)

where η_fraction_ is 0.26 (26%) for calculating the diffusion coefficient of an antibiotic in a 0.5% gel based on previous measurements obtained using a 2% gel, and η_fraction_ is 0.40 (40%) for adjusting previous measurements obtained using a 1.7% gel, and η_fraction_ is 0.50 (50%) for adjusting previous measurements obtained using a 1.5% gel. Note that these values are approximations based on experimental studies using agar gels (2). The argumentation above and using Eq. S5 leads to the D_corr_ values presented in Table S1.

**References**

1. Hou Z, An Y, Hjort K, Hjort K, Sandegren L, Wu Z. 2014. Time lapse investigation of antibiotic susceptibility using a microfluidic linear gradient 3D culture device. Lab Chip 14:3409–3418.

2. Derbyshire W, Duff ID. 1974. N.m.r. of agarose gels. Faraday Discuss Chem Soc 57:243–254.
