# Supplementary material for: A Multiplex Fluidic Chip for Rapid Phenotypic Antibiotic Susceptibility Testing
Source: mBio. 2020 Feb 25;11(1):e03109-19. doi: 10.1128/mBio.03109-19 (PMC7042698; doi:10.1128/mBio.03109-19)

**Figure S1.** **Summary of raw data (reported MIC values over time) for all analyzed strains.** Growth controls (untreated) are shown to the left.


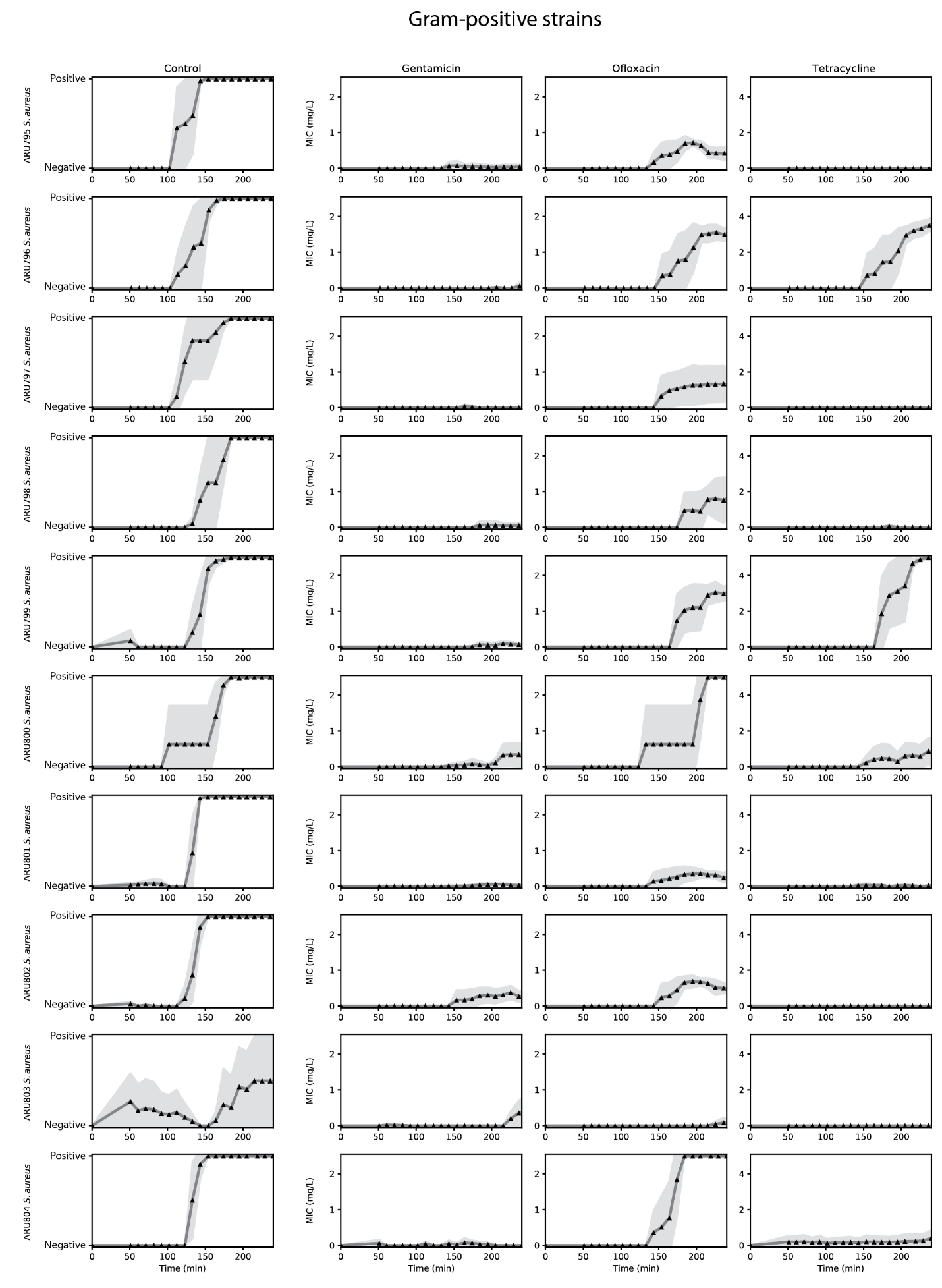


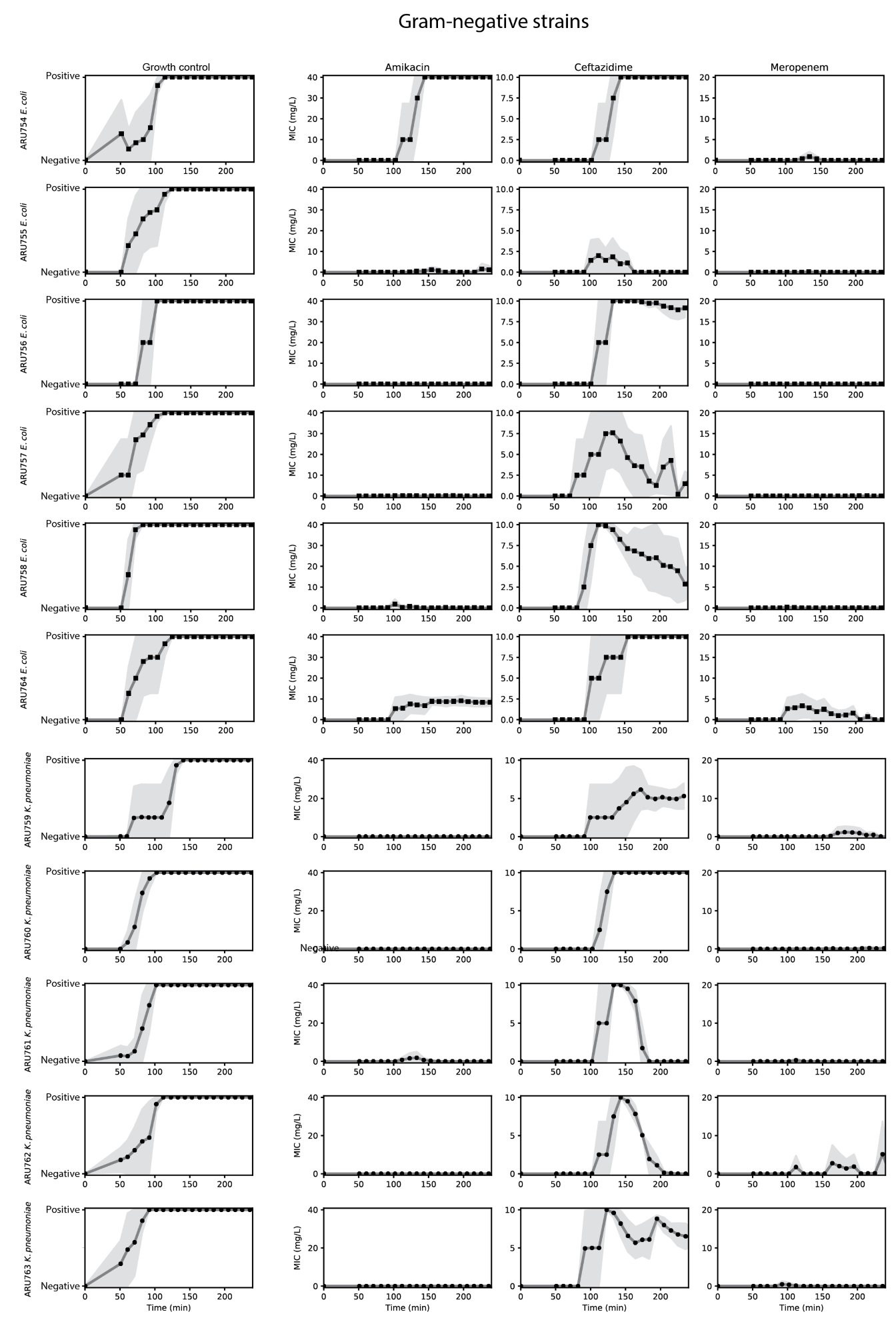

Supplement: FIG S1 [file mBio.03109-19-sf001.docx]
