# Supplementary material for: A Multiplex Fluidic Chip for Rapid Phenotypic Antibiotic Susceptibility Testing
Source: mBio. 2020 Feb 25;11(1):e03109-19. doi: 10.1128/mBio.03109-19 (PMC7042698; doi:10.1128/mBio.03109-19)
Supplement: TABLE S2 [file mBio.03109-19-st002.docx]

**Supplementary Table S2.** Summary of time to MIC and MIC values for all analyzed strains.

A. Time to MIC (TTM) and MIC values for all Gram-negative strains tested.

| **Strain** | **Rep** |  | **Control** | |  | **Amikacin** | |  | **Ceftazidime** | |  | **Meropenem** | |
| --- | --- | --- | --- | --- | --- | --- | --- | --- | --- | --- | --- | --- | --- |
|  |  |  | TTM (min) | MIC (mg/L) |  | TTM (min) | MIC (mg/L) |  | TTM (min) | MIC (mg/L) |  | TTM (min) | MIC (mg/L) |
| ARU764 | 1 |  | 105 | 1 |  | 126 | 11.97 |  | 136 | 10 |  | 136 | 4.74 |
|  | 2 |  | 123 | 1 |  | 144 | 9.40 |  | 154 | 10 |  | 144 | 0 |
|  | 3 |  | 154 | 1 |  | 174 | 9.88 |  | 174 | 10 |  | 154 | 0 |
|  | 4 |  | 102 | 1 |  | 133 | 8.27 |  | 133 | 10 |  | 133 | 5.62 |
| **Median** |  |  | 114 | 1 |  | 139 | 9.64 |  | 145 | 10 |  | 140 | 2.37 |
| **SD** |  |  | 21 | 0 |  | 18 | 1.34 |  | 16 | 0 |  | 8 | 2.61 |
|  |  |  |  |  |  |  |  |  |  |  |  |  |  |
| ARU754 | 1 |  | 137 | 1 |  | 158 | 40 |  | 158 | 10 |  | 158 | 0.00 |
|  | 2 |  | 133 | 1 |  | 154 | 40 |  | 174 | 10 |  | 133 | 0 |
|  | 3 |  | 144 | 1 |  | 164 | 40 |  | 164 | 10 |  | 144 | 0 |
|  | 4 |  | 112 | 1 |  | 133 | 40 |  | 143 | 10 |  | 112 | 0 |
| **Median** |  |  | 135 | 1 |  | 156 | 40 |  | 161 | 10 |  | 139 | 0.00 |
| **SD** |  |  | 12 | 0 |  | 12 | 0 |  | 11 | 0 |  | 17 | 0.00 |
|  |  |  |  |  |  |  |  |  |  |  |  |  |  |
| ARU755 | 1 |  | 102 | 1 |  | 102 | 0 |  | 164 | 0 |  | 102 | 0 |
|  | 2 |  | 113 | 1 |  | 113 | 0 |  | 164 | 0 |  | 113 | 0 |
|  | 3 |  | 154 | 1 |  | 164 | 0.00 |  | 185 | 0 |  | 154 | 0 |
|  | 4 |  | 133 | 1 |  | 154 | 1.77 |  | 184 | 0 |  | 143 | 0 |
| **Median** |  |  | 123 | 1 |  | 134 | 0.00 |  | 174 | 0 |  | 128 | 0.00 |
| **SD** |  |  | 20 | 0 |  | 26 | 0.77 |  | 10 | 0 |  | 21 | 0.00 |
|  |  |  |  |  |  |  |  |  |  |  |  |  |  |
| ARU756 | 1 |  | 134 | 1 |  | 134 | 0 |  | 175 | 10 |  | 134 | 0 |
|  | 2 |  | 134 | 1 |  | 134 | 0 |  | 175 | 10 |  | 134 | 0 |
|  | 3 |  | 113 | 1 |  | 113 | 0 |  | 144 | 10 |  | 113 | 0 |
|  | 4 |  | 113 | 1 |  | 113 | 0 |  | 164 | 10 |  | 113 | 0 |
| **Median** |  |  | 124 | 1 |  | 124 | 0 |  | 170 | 10 |  | 123.5 | 0 |
| **SD** |  |  | 11 | 0 |  | 11 | 0 |  | 13 | 0 |  | 10.5 | 0 |
|  |  |  |  |  |  |  |  |  |  |  |  |  |  |
| ARU757 | 1 |  | 102 | 1 |  | 123 | 0.48 |  | 257 | 2.23 |  | 257 | 2.22892 |
|  | 2 |  | 123 | 1 |  | 123 | 0.00 |  | 246 | 0 |  | 246 | 0 |
|  | 3 |  | 82 | 1 |  | 82 | 0 |  | 226 | 0 |  | 226 | 0 |
|  | 4 |  | 133 | 1 |  | 133 | 0 |  | 175 | 0 |  | 175 | 0 |
| **Median** |  |  | 113 | 1 |  | 123 | 0.00 |  | 236 | 0.00 |  | 236 | 0.00 |
| **SD** |  |  | 20 | 0 |  | 20 | 0.21 |  | 31 | 0.97 |  | 31 | 0.97 |
|  |  |  |  |  |  |  |  |  |  |  |  |  |  |
| ARU758 | 1 |  | 102 | 1 |  | 113 | 0.00 |  | 257 | 3.86 |  | 102 | 0 |
|  | 2 |  | 92 | 1 |  | 113 | 0.80 |  | 205 | 0.00 |  | 92 | 0 |
|  | 3 |  | 112 | 1 |  | 112 | 0 |  | 256 | 0.00 |  | 112 | 0 |
|  | 4 |  | 102 | 1 |  | 112 | 0 |  | 256 | 5.22 |  | 123 | 0 |
| **Median** |  |  | 102 | 1 |  | 113 | 0.00 |  | 256 | 1.93 |  | 107 | 0.00 |
| **SD** |  |  | 8 | 0 |  | 0 | 0.38 |  | 24 | 1.82 |  | 8 | 0.00 |
|  |  |  |  |  |  |  |  |  |  |  |  |  |  |
| ARU759 | 1 |  | 98 | 1 |  | 98 | 0 |  | 130 | 10.00 |  | 98 | 0 |
|  | 2 |  | 174 | 1 |  | 174 | 0 |  | 195 | 3.96 |  | 195 | 3.65 |
|  | 3 |  | 143 | 1 |  | 143 | 0 |  | 256 | 5.44 |  | 143 | 0 |
|  | 4 |  | 158 | 1 |  | 158 | 0 |  | 199 | 3.53 |  | 158 | 0 |
| **Median** |  |  | 151 | 1.00 |  | 151 | 0 |  | 197 | 4.70 |  | 151 | 0.00 |
| **SD** |  |  | 28 | 0.00 |  | 28 | 0 |  | 45 | 2.56 |  | 35 | 1.58 |
|  |  |  |  |  |  |  |  |  |  |  |  |  |  |
| ARU760 | 1 |  | 124 | 1 |  | 124 | 0 |  | 156 | 10 |  | 124 | 0 |
|  | 2 |  | 135 | 1 |  | 135 | 0 |  | 156 | 10 |  | 135 | 0 |
|  | 3 |  | 123 | 1 |  | 123 | 0 |  | 143 | 10 |  | 123 | 0 |
|  | 4 |  | 112 | 1 |  | 123 | 0 |  | 133 | 10 |  | 133 | 0.08 |
| **Median** |  |  | 124 | 1 |  | 124 | 0.00 |  | 150 | 10 |  | 129 | 0.00 |
| **SD** |  |  | 8 | 0 |  | 5 | 0.00 |  | 10 | 0 |  | 5 | 0.03 |
|  |  |  |  |  |  |  |  |  |  |  |  |  |  |
| ARU761 | 1 |  | 133 | 1 |  | 133 | 0 |  | 195 | 0 |  | 133 | 0 |
|  | 2 |  | 133 | 1 |  | 133 | 0 |  | 195 | 0 |  | 133 | 0 |
|  | 3 |  | 112 | 1 |  | 112 | 0 |  | 195 | 0 |  | 112 | 0 |
|  | 4 |  | 112 | 1 |  | 153 | 1.12 |  | 205 | 0 |  | 123 | 0 |
| **Median** |  |  | 123 | 1 |  | 133 | 0.00 |  | 195 | 0 |  | 128 | 0.00 |
| **SD** |  |  | 11 | 0 |  | 14 | 0.49 |  | 4 | 0 |  | 9 | 0.00 |
|  |  |  |  |  |  |  |  |  |  |  |  |  |  |
| ARU762 | 1 |  | 133 | 1 |  | 133 | 0 |  | 205 | 0 |  | 133 | 0 |
|  | 2 |  | 112 | 1 |  | 112 | 0 |  | 225 | 0 |  | 123 | 0 |
|  | 3 |  | 133 | 1 |  | 133 | 0 |  | 205 | 0 |  | 133 | 0 |
|  | 4 |  | 143 | 1 |  | 143 | 0 |  | 246 | 0 |  | 143 | 0 |
| **Median** |  |  | 133 | 1 |  | 133 | 0 |  | 215 | 0.00 |  | 133 | 0.00 |
| **SD** |  |  | 11 | 0 |  | 11 | 0 |  | 17 | 0.00 |  | 7 | 0.00 |
|  |  |  |  |  |  |  |  |  |  |  |  |  |  |
| ARU763 | 1 |  | 92 | 1 |  | 92 | 0 |  | 256 | 5.82 |  | 92 | 0 |
|  | 2 |  | 92 | 1 |  | 92 | 0 |  | 256 | 8.80 |  | 112 | 0.44 |
|  | 3 |  | 123 | 1 |  | 123 | 0 |  | 256 | 3.25 |  | 123 | 0 |
|  | 4 |  | 123 | 1 |  | 123 | 0 |  | 256 | 6.89 |  | 123 | 0 |
| **Median** |  |  | 108 | 1 |  | 108 | 0.00 |  | 256 | 6.36 |  | 118 | 0.00 |
| **SD** |  |  | 16 | 0 |  | 16 | 0.00 |  | 0 | 2.00 |  | 13 | 0.19 |

B. Time to MIC (TTM) and MIC values for all Gram-positive strains tested.

| **Strain** | **Rep** |  | **Control** | |  | **Gentamicin** | |  | **Ofloxacin** | |  | **Tetracycline** | |
| --- | --- | --- | --- | --- | --- | --- | --- | --- | --- | --- | --- | --- | --- |
|  |  |  | TTM (min) | MIC (mg/L) |  | TTM (min) | MIC (mg/L) |  | TTM (min) | MIC (mg/L) |  | TTM (min) | MIC (mg/L) |
| ARU795 | 1 |  | 184 | 1 |  | 215 | 0 |  | 205 | 0.84 |  | 215 | 0 |
|  | 2 |  | 143 | 1 |  | 153 | 0.31 |  | 153 | 0.79 |  | 174 | 0 |
|  | 3 |  | 155 | 1 |  | 186 | 0 |  | 165 | 0.75 |  | 186 | 0.000 |
|  | 4 |  | 175 | 1 |  | 175 | 0.02 |  | 175 | 0.49 |  | 207 | 0 |
| **Median** |  |  | 165 | 1 |  | 181 | 0.01 |  | 170 | 0.77 |  | 197 | 0.0000 |
| **SD** |  |  | 16 | 0 |  | 22 | 0.13 |  | 19 | 0.14 |  | 16 | 0.0000 |
|  |  |  |  |  |  |  |  |  |  |  |  |  |  |
| ARU796 | 1 |  | 195 | 1 |  | 225 | 0 |  | 195 | 1.16 |  | 297 | 4.83 |
|  | 2 |  | 154 | 1 |  | 184 | 0 |  | 225 | 1.56 |  | 195 | 3.38 |
|  | 3 |  | 208 | 1 |  | 208 | 0.06 |  | 208 | 1.41 |  | 269 | 4.12 |
|  | 4 |  | 177 | 1 |  | 208 | 0.00 |  | 218 | 1.81 |  | 208 | 2.53 |
| **Median** |  |  | 186 | 1 |  | 208 | 0.00 |  | 213 | 1.49 |  | 239 | 3.75 |
| **SD** |  |  | 20 | 0 |  | 15 | 0.03 |  | 11 | 0.24 |  | 42 | 0.85 |
|  |  |  |  |  |  |  |  |  |  |  |  |  |  |
| ARU797 | 1 |  | 164 | 1 |  | 195 | 0 |  | 205 | 0.55 |  | 195 | 0 |
|  | 2 |  | 164 | 1 |  | 205 | 0 |  | 164 | 0.34 |  | 195 | 0 |
|  | 3 |  | 215 | 1 |  | 246 | 0 |  | 246 | 0.00 |  | 246 | 0 |
|  | 4 |  | 153 | 1 |  | 195 | 0 |  | 153 | 1.31 |  | 184 | 0 |
| **Median** |  |  | 164 | 1 |  | 200 | 0 |  | 185 | 0.45 |  | 195 | 0 |
| **SD** |  |  | 24 | 0 |  | 21 | 0 |  | 37 | 0.48 |  | 24 | 0 |
|  |  |  |  |  |  |  |  |  |  |  |  |  |  |
| ARU798 | 1 |  | 215 | 1 |  | 246 | 0 |  | 277 | 0.25 |  | 246 | 0 |
|  | 2 |  | 215 | 1 |  | 246 | 0 |  | 236 | 0.79 |  | 246 | 0 |
|  | 3 |  | 184 | 1 |  | 195 | 0.26 |  | 205 | 0.44 |  | 225 | 0 |
|  | 4 |  | 184 | 1 |  | 215 | 0.00 |  | 195 | 1.21 |  | 215 | 0 |
| **Median** |  |  | 200 | 1 |  | 231 | 0.00 |  | 221 | 0.61 |  | 236 | 0 |
| **SD** |  |  | 16 | 0 |  | 22 | 0.11 |  | 32 | 0.37 |  | 13 | 0 |
|  |  |  |  |  |  |  |  |  |  |  |  |  |  |
| ARU799 | 1 |  | 215 | 1 |  | 215 | 0.19 |  | 215 | 1.03 |  | 256 | 5 |
|  | 2 |  | 174 | 1 |  | 205 | 0 |  | 174 | 1.54 |  | 174 | 5 |
|  | 3 |  | 184 | 1 |  | 184 | 0.20 |  | 246 | 1.48 |  | 246 | 5 |
|  | 4 |  | 184 | 1 |  | 184 | 0.06 |  | 184 | 1.54 |  | 266 | 5 |
| **Median** |  |  | 184 | 1 |  | 195 | 0.12 |  | 200 | 1.51 |  | 251 | 5.00 |
| **SD** |  |  | 15 | 0 |  | 13 | 0.09 |  | 28 | 0.21 |  | 36 | 0.00 |
|  |  |  |  |  |  |  |  |  |  |  |  |  |  |
| ARU800 | 1 |  | 215 | 1 |  | 246 | 0.87 |  | 215 | 2.5 |  | 246 | 0.00 |
|  | 2 |  | 215 | 1 |  | 215 | 0.25 |  | 215 | 2.5 |  | 225 | 0.73 |
|  | 3 |  | 205 | 1 |  | 205 | 0.034 |  | 236 | 2.5 |  | 246 | 0.00 |
|  | 4 |  | 133 | 1 |  | 153 | 0.167 |  | 143 | 2.5 |  | 266 | 2.01 |
| **Median** |  |  | 210 | 1 |  | 210 | 0.21 |  | 215 | 2.50 |  | 246 | 0.37 |
| **SD** |  |  | 34 | 0 |  | 33 | 0.32 |  | 35 | 0.00 |  | 14 | 0.82 |
|  |  |  |  |  |  |  |  |  |  |  |  |  |  |
| ARU801 | 1 |  | 184 | 1 |  | 215 | 0.000 |  | 225 | 0.30 |  | 215 | 0 |
|  | 2 |  | 164 | 1 |  | 164 | 0.05 |  | 174 | 0.62 |  | 164 | 0.18 |
|  | 3 |  | 174 | 1 |  | 205 | 0.08 |  | 215 | 0.23 |  | 205 | 0 |
|  | 4 |  | 174 | 1 |  | 225 | 0 |  | 205 | 0.47 |  | 205 | 0 |
| **Median** |  |  | 174 | 1.00 |  | 210 | 0.02 |  | 210 | 0.38 |  | 205 | 0.00 |
| **SD** |  |  | 7 | 0.00 |  | 23 | 0.03 |  | 19 | 0.15 |  | 26 | 0.09 |
|  |  |  |  |  |  |  |  |  |  |  |  |  |  |
| ARU802 | 1 |  | 174 | 1 |  | 195 | 0.16 |  | 174 | 0.59 |  | 205 | 0 |
|  | 2 |  | 174 | 1 |  | 174 | 0.68 |  | 174 | 0.94 |  | 205 | 0 |
|  | 3 |  | 174 | 1 |  | 205 | 0.11 |  | 215 | 0.54 |  | 205 | 0 |
|  | 4 |  | 184 | 1 |  | 184 | 0.28 |  | 195 | 0.64 |  | 215 | 0 |
| **Median** |  |  | 174 | 1 |  | 190 | 0.22 |  | 185 | 0.61 |  | 205 | 0.00 |
| **SD** |  |  | 4 | 0 |  | 12 | 0.22 |  | 17 | 0.15 |  | 4 | 0.00 |
|  |  |  |  |  |  |  |  |  |  |  |  |  |  |
| ARU803 | 1 |  | 297 | 0.67 |  | 297 | 0.70 |  | 297 | 0.52 |  | 297 | 0 |
|  | 2 |  | 225 | 1 |  | 277 | 0.86 |  | 256 | 0.00 |  | 256 | 0 |
|  | 3 |  | 298 | 0 |  | 298 | 0 |  | 298 | 0 |  | 298 | 0 |
|  | 4 |  | 246 | 1 |  | 266 | 0.53 |  | 287 | 0 |  | 277 | 0 |
| **Median** |  |  | 272 | 0.84 |  | 287 | 0.61 |  | 292 | 0.00 |  | 287 | 0.00 |
| **SD** |  |  | 32 | 0.41 |  | 14 | 0.32 |  | 17 | 0.22 |  | 17 | 0.00 |
|  |  |  |  |  |  |  |  |  |  |  |  |  |  |
| ARU804 | 1 |  | 184 | 1 |  | 215 | 0 |  | 184 | 2.5 |  | 215 | 0.00 |
|  | 2 |  | 164 | 1 |  | 164 | 0.30 |  | 215 | 2.5 |  | 195 | 0.00 |
|  | 3 |  | 174 | 1 |  | 205 | 0 |  | 174 | 2.5 |  | 205 | 0 |
|  | 4 |  | 174 | 1 |  | 195 | 0.12 |  | 195 | 2.5 |  | 185 | 0.71 |
| **Median** |  |  | 174 | 1 |  | 200 | 0.06 |  | 190 | 2.50 |  | 200 | 0.00 |
| **SD** |  |  | 7 | 0 |  | 19 | 0.12 |  | 15 | 0.00 |  | 11 | 0.31 |
